# Research of Binary and Ternary Composites Based on Selected Aliphatic or Aliphatic–Aromatic Polymers, 5CB or SWCN toward Biodegradable Electrodes

**DOI:** 10.3390/ma13112480

**Published:** 2020-05-29

**Authors:** Patryk Fryń, Beata Jewłoszewicz, Krzysztof Artur Bogdanowicz, Wojciech Przybył, Agnieszka Gonciarz, Robert Pich, Monika Marzec, Agnieszka Iwan

**Affiliations:** 1 Institute of Physics, Jagiellonian University, 30-348 Krakow, Poland; patryk.fryn@doctoral.uj.edu.pl; 2 Military Institute of Engineer Technology, Obornicka 136 Str., 50-961 Wroclaw, Poland; jewloszewicz@witi.wroc.pl (B.J.); bogdanowicz@witi.wroc.pl (K.A.B.); przybyl@witi.wroc.pl (W.P.); 3 Faculty of Security and Safety Research, General Tadeusz Kosciuszko Military University of Land Forces, Czajkowskiego 109 Str., 51-147 Wroclaw, Poland; agnieszka.gonciarz@awl.edu.pl (A.G.); robert.pich@awl.edu.pl (R.P.)

**Keywords:** single walled carbon nanotubes, L,D-poly(lactic acid), polycaprolactone, Ecoflex^®^, 5CB, flexible substrate

## Abstract

The main goal of this paper was to study the optical, electrical, and thermal properties of hybrid composites based on biodegradable polymers (L,D-poly(lactic acid), polycaprolactone or Ecoflex^®^), single walled carbon nanotubes (SWCN), and 4′-pentyl-4-biphenylcarbonitrile (5CB). The biodegradable polymers’ binary and ternary compositions were analyzed in detail by ultraviolet and visible (UV–Vis) spectroscopy taking into consideration their chemical structure and interactions with 5CB and SWCN. Differential scanning calorimetry (DSC) studies of the created hybrid layers showed thermal stability and changes in glass transition temperature and melting point in comparison to neat polymers, depending on the chemical structure of the polymer used and the type of composition. Morphology of the created layers were investigated by atomic force and polarizing microscopy. The static contact angle measurements of a water drop showed that all of the neat polymer layers were hydrophobic with angle values ranging from 108° to 115°. In addition, in the case of the Ecoflex^®^ layers, both with and without additives, a rapid sorption of the deposited water drop was observed. Finally, a simple device with poly(ethylene terephthalate) (PET)/indium tin oxide (ITO)/poly(3,4-ethylenedioxythiophene):poly(styrenesulfonate) (PEDOT:PSS)/poly [[4,8-bis[(2-ethylhexyl)oxy]benzo[1,2-b:4,5-b′]dithiophene-2,6-diyl][3-fluoro-2-[(2-ethylhexyl)carbonyl]thieno[3,4-b]thiophenediyl]] (PTB7): [6,6]-phenyl-C71-butyric acid methyl ester (PC_70_BM)/Ag/biodegradable polymer:SWCN architecture was constructed and tested using an infrared (IR) thermographic camera to investigate the surface defects on the created hybrid layers. Increasing the SWCN admixture from 0.01 to 0.5% significantly improved the conductivity only in the case of L,D-poly(lactic acid):SWCN (10:0.5), for which above 5 V, a current with a resistance of 3030.7 Ω could be measured. In order to use the created layers as flexible electrodes, the first experiments were carried out with an admixture of SWCN and poly(3,4-ethylenedioxythiophene):poly(styrenesulfonate) as conductive compounds.

## 1. Introduction

**Scheme 1:**
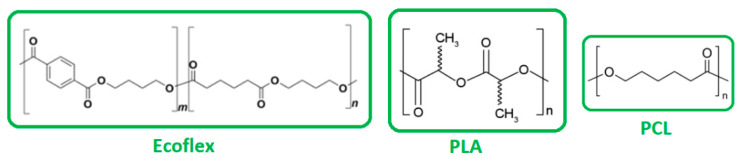
Chemical structures of Ecoflex^®^, polylactic acid (PLA), and polycaprolactone (PCL).

Recycling and biodegradability are matters of growing concern in the context of designing new functional materials and their use in modern electronic devices. The ecological aspect of the proposed solutions is important from the point of view of practical applications and the longstanding usage of the produced devices. Among the various biodegradable polymers, special attention has been paid to poly(lactic acid), polycaprolactone, or Ecoflex^®^ as a biodegradable substrate [1,2,3,4,5,6,7,8]. Briefly, composites of poly-butylene adipate-co-terephthalate (PBAT, brand name Ecoflex^®^) with carbon nanotubes have been tested in supercapacitors and skin-mountable strain sensors [9,10,11]. In our previous work, we investigated compositions based on Ecoflex^®^ and graphene oxide toward organic photovoltaics [12]. The chemical structure of Ecoflex^®^ is presented in Scheme 1. Our idea of testing Ecoflex^®^ as a flexible substrate was based on properties such as excellent mechanical properties, strong interfacial bonding between the carbon nanotubes and the Ecoflex^®^, and water and weather resistance, making it suitable for long-term applications [10,13].

We also carefully investigated the influence of SWCN and 5CB on the selected properties of L,D-PLA to find the best weight ratio of additives to L,D-PLA [14]. Our study showed that a 0.01% concentration of SWCN in L,D-PLA gave a translucent substrate; however, it did not exhibit sufficient conductivity. An increase in the amount of SWCN up to 5% in the L,D-PLA matrix caused an increase in conductivity to about 1.01 × 10^3^ S/m; however, the films were not fully translucent [14]. In our last work [15], we found that the best substrate flexibility and short-term strength were obtained for L,D-PLA:5CB with ratio of 10:1 *w*/*w*. Increasing the proportion of 5CB above 30% reduced the strength and elasticity of the film. In turn, the addition of SWCN to binary compositions based on L,D-PLA:5CB caused an almost twofold increase in the strength and an almost tenfold decrease in their elasticity. Our previous studies showed that the best electrical conductivity is for L,D-PLA:5CB:SWCN with a ratio of 10:1:0.5 *w*/*w*/*w* [15]. Moreover, other scientists have also investigated PLA in compositions with carbon nanotubes [16,17,18,19,20,21,22,23]. The chemical structure of PLA is presented in Scheme 1. L,D-PLA is known as an environmentally friendly polymer (is compostable and can be obtained from renewable resources) showing a high elastic modulus and a high transparency [6,7]. Additionally, L,D-PLA has a low glass transition temperature, low thermal stability, high brittleness, and low crystallization rate. 

Polycaprolactone (PCL) is known to have a low melting point (about 60 °C) and a glass transition temperature of about −60 °C, however, its characteristics depend on the molecular weight. PCL degrades slowly by the hydrolysis process, caused by its high crystallinity and hydrophobic nature. Among the various biological, ecological, and medical applications, PCL has been implemented as implantable biomaterials, biodegradable materials, and microparticles for drug delivery [24,25,26,27,28,29,30,31]. Additionally, the effect of carbon nanotubes and silver nanoparticles on the biological properties of PCL were tested [30]. It was found that silver nanoparticles simplify the electron transfer in the PCL matrix in the presence of SWCN. In [31], wherein the PCL:SWCN composition was studied, a higher conductive network efficiency was observed for a SWCN concentration lower than 1 wt %. The chemical structure of PCL is presented in Scheme 1.

Carbon nanotubes, as one-dimensional nanomaterials, are still a particular area of interest because of the rare combination of mechanical, thermal, and electrical properties. They are still regarded as promising candidates for the replacement of transparent conducting oxides (e.g., indum tin oxide—ITO or fluorine-doped tin oxide—FTO) in the developing field of so called plastic electronics, due to their transparency in thin films, high electrical conductivity, excellent mechanical properties, characteristic flexibility, and the potential for roll-to-roll processing [32,33,34]. Carbon nanotubes were investigated as transparent anodes in polymer solar cells (standard and inverted architecture) to replace the expensive, stiff, and brittle ITO substrate [35]. In our research, we used metallic, conductive nanotubes (armchair type). The addition of this type of nanotube causes hybrid materials to become conductive, which was our goal. 

It is known that the conductivity of a polymer composite containing SWCN depends on the orientation of carbon nanotubes in a polymer matrix. The orientation can usually be induced by extrusion and can show improved conductivity in a specific direction, mainly in-plane. In our case, the formation of thin films out of the L,D-PLA used by us was difficult; in order to keep good biodegradability, the use of a very long chain polymer was not an option. In our study, we used a polymer with approximately 101 repetitive units (average mass = 7274 g/mol), which resulted in a material possessing poor thermal processability. This was the reason for not including the orientation of SWCN in this work.

To improve the resistance value of composites from SWCN, for example, the addition of a metallic grid (e.g., Ag) is used, which is one of the approaches in obtaining good optical absorption (SWCN) and high conductivity (metallic grid) [36,37]. 

Given ours and other previous works, the main idea of this work was to make our own comparative studies of three biodegradable polymers as a flexible substrate containing liquid crystal or/and single walled carbon nanotubes toward the selection of the best flexible substrate (flexible anode or cathode or encapsulation material) for organic devices. Hence, in this work, the effect of 4′-pentyl-4-biphenylcarbonitrile (5CB) as a liquid crystal and conductive single walled carbon nanotube (SWCN) in biodegradable polymers like Ecoflex^®^, polycaprolactone (PCL) and polylactide (L,D-PLA) on the selected properties of the created hybrid layers for potential organic device applications were examined. Our study included four main factors such as the influence of:

(i) the type of biodegradable polymers;
(ii) the type of composition: binary or ternary compositions; and
(iii) the amount of SWCN

on the transparency, morphology, thermal and chemical stability, wetting properties, and electrical behavior of created binary and ternary hybrid composites.

## 2. Experimental 

### 2.1. Materials 

The 4′-pentyl-4-biphenylcarbonitrile (5CB) and single walled carbon nanotubes (SWCN) were used as received from Sigma-Aldrich. The SWCN used had an average diameter of 0.84 nm, median length 1 µm, ≥ 95% carbon basis (≥ 99% as carbon nanotubes). L,D-poly(lactic acid) (L,D-PLA) was used as received from Galactic. Biodegradable aliphatic-aromatic copolyester (produced by BASF under the commercial mark Ecoflex^®^) was used as received. PEDOT:PSS Al 4083:poly(3,4-ethylenedioxythiophene):poly(styrenesulfonate) was received from Ossila Ltd. ITO coated PET (poly(ethylene terephthalate)) with resistance 10^−14^ Ω/square, and transparency 70–72% was provided by 3D-nano (Kraków, Poland).

#### 2.1.1. Preparation of Solutions 

**Scheme 2:**
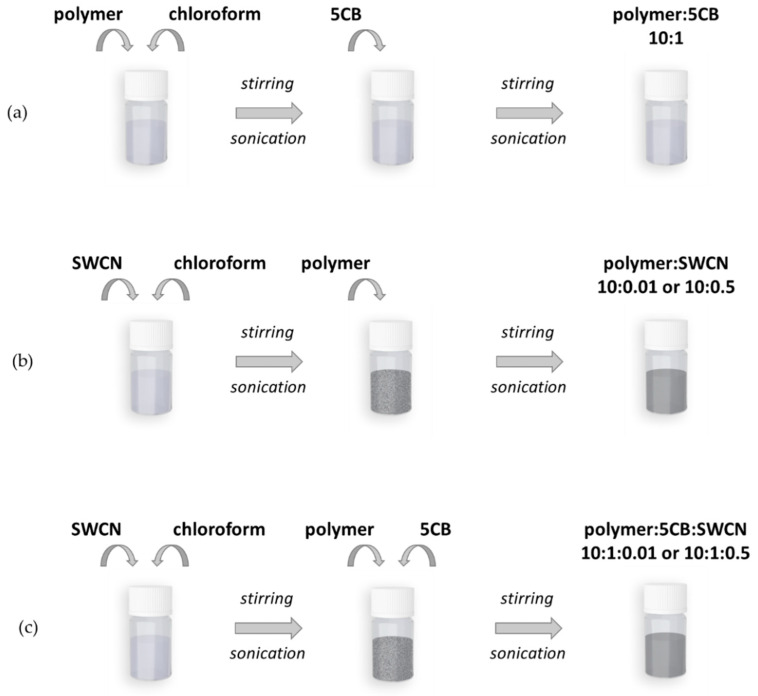
Scheme of the solution preparation of the polymer:5CB (**a**), polymer:SWCN (**b**), and polymer:5CB:SWCN (**c**).

Solutions of the polymer (L,D-PLA, PCL, or Ecoflex^®^) and its mixture with 5CB liquid crystal or single-walled carbon nanotubes (SWCN) were prepared as described below: appropriate amounts of polymer and 5CB in a ratio of 10:1 (*w*/*w*) or polymer and SWCN in a ratio of 10:0.01 (*w*/*w*) and 10:0.5 (*w*/*w*) were poured into 4 mL of high purity chloroform. Each solution was mixed on a magnetic stirrer for at least 2 h, followed by sonication for 40 min, 10 min, and 20 min for L,D-PLA, PCL, and Ecoflex^®^, respectively. Solutions containing SWCN were used immediately after preparation because of their instability over time. Details of the optimization of the preparation of solutions are presented in Scheme 2.

#### 2.1.2. Preparation of Hybrid Layers 

**Scheme 3:**
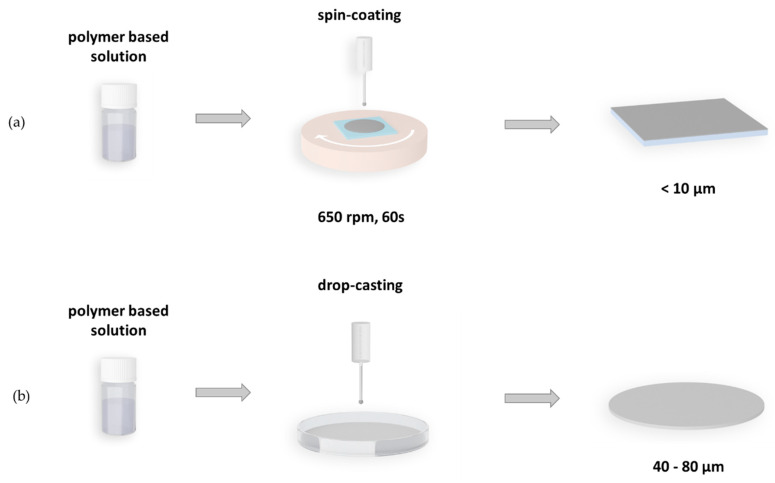
Scheme of layer preparation using the spin coating (**a**) and drop casting (**b**) techniques.

The solutions for the layer preparation were prepared as described above. The layers were obtained by two methods: spin-coating and drop casting (on a small glass substrate and on a Petri glass). In the spin-coating method, a small amount of mixture in chloroform (c.a. 0.5 mL) were dropped on a rotating glass substrate (c.a. 1 × 1.5 cm^2^) at 650 rotations per minute for 60 s. During this procedure, the chloroform evaporated and the thin hybrid layers were obtained. The layer thickness was measured with electric calipers with accuracy of ±10 µm and were found in the range of 40 to 80 µm for the drop-casting method. The layers produced by the spin-coating method were too thin (thicknesses in the range of caliper error), hence were not separated from the glass substrate. Details of the optimized spin coating and drop-casting techniques for the investigated compositions are presented in Scheme 3. 

### 2.2. Characterization of Methods

Layer topography by atomic force microscopy (AFM) imaging was performed at room temperature by using an Agilent 5500 microscope (Agilent Technologies, Santa Clara, California, CA, USA) working in non-contact mode. Parameters such as setpoint, proportional gain, integral gain, and speed were adjusted to each measurement to obtain the best possible images. Topography images were collected at several randomly chosen areas for each sample to make sure that the presented results were reliable [15]. Image processing was done using the Gwyddion [1].

Differential scanning calorimetry (DSC) measurements were done using a Perkin Elmer DSC 8000 calorimeter (PerkinElmer Inc., Waltham, Massachusetts, MA, USA) to find the transition temperatures of the new created hybrid materials. The samples of ca. 10 mg were placed into 30 μL aluminum crucibles and tightly closed with a press. The measurements were performed with a 10 °C/min rate both on heating and cooling. At least three cycles of heating and cooling were done for each sample to ensure the repeatability of the results [15].

Texture observations were performed using a Nikon Eclipse LV100POL polarizing microscope (NIKON Inc., Tokyo, Japan) equipped with a Fine Instruments WTMS-14C heating stage. The images were registered by a computer-controlled camera (Canon EOS 600D). A small piece of each created layer (c.a. 1 cm^2^) was placed between the glass substrate and the cover glass and put inside the heating stage [15].

The transmission UV–Vis spectra of the layers prepared by the spin-coating method were acquired using an V-570 Jasco UV–Vis spectrophotometer (Jasco Inc., Easton, MD, USA) in the measuring range of 190–1100 nm with the scan rate 100 nm/min, with correction on air. The transmission UV–Vis spectra of the layers prepared by the drop-casting method were acquired using an A360 UV–Vis spectrophotometer (AOR Instruments, Shanghai, China) with an interval of 0.2 nm and medium scan speed [15]. The absorption UV–Vis spectra of all polymers and hybrid compositions in chloroform solution were obtained by using an UV–Vis Agilent Cary 300 (Agilent Technologies, Santa Clara, CA, United States).

A contact angle goniometer (Ossila, UK) coupled with a computer system served as the basic diagnostic tool that enabled contact angle measurements by analyzing the shape of the drop after wetting the surface with water. The drop images were recorded up to 10 s, counting from the moment the drop touched the surface. The volume of a drop dosed with a syringe was approximately 5 μL. The angle between the tangent to the surface of the drop placed on the solid at the point of contact of three phases: solid, liquid, and gas was measured to assess the hydrophobic behavior [38].

Thermal behavior was investigated using a thermographic camera (VIGOcam v50, VIGO System S.A, Ożarów Mazowiecki, Poland) by applying bias voltage between 0 and 10 V and using a multichannel potentiostat-galvanostat (PGStat Autolab M101, Barendrecht, Metrohm, Netherlands). The experiment was designed as follows: the potential was changed from 0 V to 10 V with a step of 0.5 V with three minutes for each voltage. The current response was recorded during this three-minute-interval and each step was separated with a 10 s window when the IR image was collected, while the current was still passing through the sample [15]. Both camera and power source were digitally controlled.

## 3. Results and Discussion 

In the first step of our work, we investigated the influence of 5CB and SWCN on the selected properties of biodegradable polymers such as L,D-poly(lactic acid) (L,D-PLA), polycaprolactone (PCL), and Ecoflex^®^. For this reason, binary and ternary compositions were created and investigated. Based on our previous results [15], we chose the weight ratios of polymer:5CB, polymer:SWCN, and polymer:5CB:SWCN in the created composites as 10:1, 10:0.01, and 10:1:0.01, respectively.

### 3.1. Thermal and Microscopic Studies

**Figure 1:**
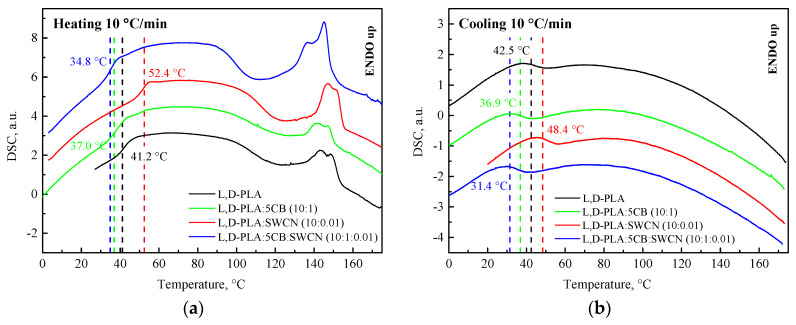
Differential scanning calorimetry (DSC) curves with glass transition (T_g_) registered during heating (**a**) and cooling (**b**) for the created hybrid layers and pure L,D-poly(lactic acid).

The transition temperatures and enthalpies of the investigated polymers and compositions determined by differential scanning calorimetry (DSC) are presented in Table 1. DSC thermograms of the L,D-PLA without and with 5CB, SWCN, and 5CB:SWCN, as an example, obtained during heating and cooling, under a N_2_ atmosphere, are shown in Figure 1. Results of the DSC measurements for other polymers and their compositions are presented in Supplementary Materials Figure S1. As one can see, the addition of 5CB and/or SWCN does not change the character of the phase transition in each studied hybrid layers. Small shifts in the transition temperatures occurred for all the hybrid layers and were the most noticeable for PCL (see T_onset_ in Table 1). 

All investigated polymers and hybrid compositions give, under the second heating and cooling cycles at 10 °C/min rate, various DSC profiles with melting/crystallization T_onset_ and glass transition T_g_ temperatures more or less visible. These findings clearly indicate their semi-crystalline nature. The glass transition temperature T_g_ of the investigated polymers was found for L,D-PLA during heating and cooling and for Ecoflex^®^ only during cooling (see Table 1 and Figure 1 and Figure S1). It is obvious that they depend on the structure of the polymers. First, we noticed that Ecoflex^®^ showed significantly higher T_g_, consistent with their more rigid chains. The addition of 5CB slightly decreased the T_g_ of L,D-PLA and Ecoflex^®^. The opposite behavior was found for compositions of polymer:SWCN. The addition of SWCN increased the Tg value for L,D-PLA and Ecoflex^®^. Quite different behavior, depending on the polymer used, was found for the ternary compositions. In the case of L,D-PLA, the addition of 5CB:SWCN decreased the value of T_g_ compared to the pure polymer and binary compositions. Peculiar behavior was observed for Ecoflex^®^:5CB:SWCN compositions when considering the value of T_g_. In this case, the value of T_g_ for ternary composition was almost identical as that for the pure polymer. In our opinion, this behavior can be explained by the fact that 5CB probably modified the morphology of the polymer (to make it more homogeneous with a special track for SWCN) and probably simplified the transfer of electrons in Ecoflex^®^ due to the presence of SWCN.

**Figure 2:**
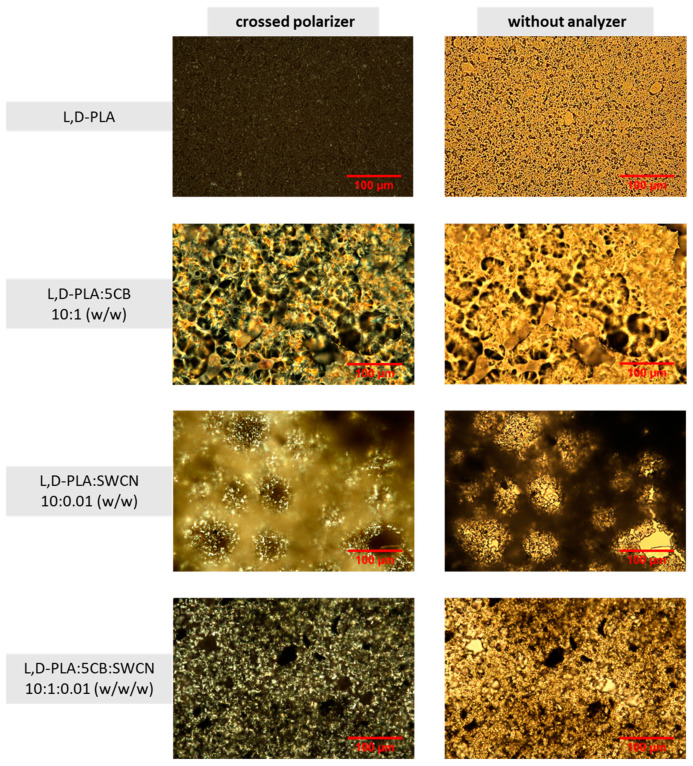
Optical textures of pure L,D-PLA and its hybrid layers registered with crossed polarizers and without an analyzer.

All optical textures were registered at room temperature under a polarizing microscope with crossed polarizers as well as without an analyzer (with one polarizer). The microphotographs taken for the pure polymers and created hybrid layers are presented in Figure 2 and Figures S2 and S3 in the Supplementary Materials. As one can see, the pure L,D-PLA layer is dark, while the admixtures changed them significantly (Figure 2). The admixture of 5CB makes the texture colored, while after the addition of SWCN, it becomes brighter. Ternary hybrid layers were brighter and more colored when compared with the layer of pure L,D-PLA.

In the case of PCL, the addition of 5CB only makes the texture darker, as compared to the pure PCL layer, but the SWCN admixture completely changed the textures (Figure S2). A similar influence of the admixtures was observed for the layers based on Ecoflex^®^ (Figure S3). There was no significant differences between the layers of neat Ecoflex^®^ and with the addition of 5CB, while the SWCN admixture made it significantly different. Summarizing the texture observations by POM, the admixture of SWCN had a dominant impact on the morphology of the created hybrid layers of all of the studied polymers.

Moreover, simple optical microscope imaging was also done for all of the prepared samples (see Figure S4). The most homogenous structure was observed for L,D-PLA (Figure S4A). The presence of 5CB in the proportion 1:10 to the polymer did not significantly change the registered images (Figure S4D). The structure of the film containing 0.01 *w*/*w* of SWCN revealed a uniform dispersion of small nanotube domains within the polymer matrix (Figure S4G). As the amount of the SWCN increased, the film became less transparent to transmitted light, and the zone dominated by carbon nanoparticles increased (Figure S4J). Films made of pure PCL demonstrated a grain-like structure (Figure S4B), which in the presence of 5CB was characterized by more dispersed and smaller domains (Figure S4E). On the other hand, the addition of SWCN to PCL resulted in a heterogeneous texture with visible zones of agglomerated nanotubes (Figure S4H,K). The optical micrographs for samples based on pure Ecoflex^®^ showed a homogenous structure (Figure S4C), while the addition of any additive caused a large dispersion of black spots (Figure S4F,I), which in the case of carbon nanotubes tend to form agglomerates (Figure S4I).

**Figure 3:**
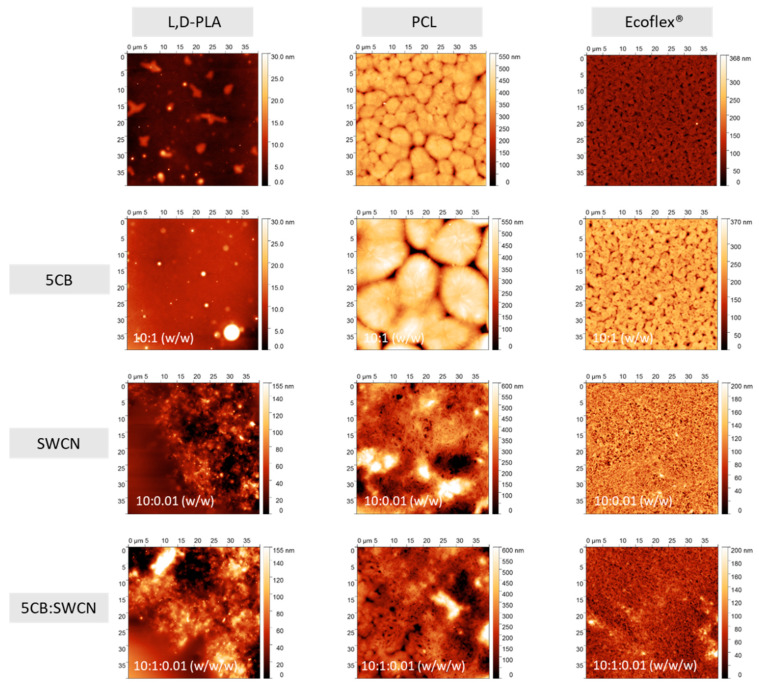
Atomic force microscopy (AFM) images of the pure studied polymers, binary, and ternary hybrid layers.

The influence of the SWCN and/or 5CB on the surface topography of the biodegradable polymers was additionally investigated by the AFM method (Figure 3). The morphology of the pure L,D-PLA surface was quite smooth and continuous with some irregular elevations up to 30 nm. PCL created regular mounds on the surface, one next to the others. Ecoflex^®^ has a tendency to create an oval random coil of a polymer chain. The addition of 5CB (10:1 *w*/*w*) changed the topography by making the structures bigger and higher, but did not change the general character of the surface, while the influence of SWCN was the most noticeable. In the case of PCL and Ecoflex^®^, both polymers covered the carbon nanotubes and the coating was larger for PCL than for Ecoflex^®^, which is clearly visible in Figure 3. On the other hand L,D-PLA did not cover the carbon nanotubes as effectively as PCL and Ecoflex^®^, and it is possible to notice single 1 μm length straight structures that came from the carbon nanotubes. No significant changes between hybrid layers with carbon nanotubes and with or without 5CB were observed for all three studied polymers.

The roughness parameters such as (Sa, Sq) for the investigated surfaces are presented in Table 2. The presented parameters are defined in Equations (1) and (2):

(1) $$S_{a}=\frac{1}{MN}\overset{M-1}{\underset{k=0}{\sum }}\overset{N-1}{\underset{l=0}{\sum }}\left|z\left(x_{k},y_{l}\right)\right|$$

(2) $$S_{q}=\sqrt{\frac{1}{MN}\overset{M-1}{\underset{k=0}{\sum }}\overset{N-1}{\underset{l=0}{\sum }}{\left[z\left(x_{k},y_{l}\right)\right]}^{2}}$$

where *M* and *N* are the total number of pixels in the analyzed scan; *k* and *l* are coordinates of a given pixel; and *z* (*x_k_*, *y_i_*) is the height of the surface at a given coordinate. To determine the surface parameters of the created layers, the most representative area of 20 μm × 20 μm, from each of the AFM images presented in Figure 3, was selected for roughness analysis.

The AFM study showed that all of the studied polymers exhibited various morphology depending on the mixture composition. The mixture L,D-PLA with 5CB exhibited a root-mean-square (Sq) roughness value of 1.44 nm, whereas the L,D-PLA:SWCN composition ca. 18.63 nm. The ternary mixture based on L,D-PLA exhibited the highest value of Sq (28.18 nm) compared to the binary mixture and pure L,D-PLA. The polymer L,D-PLA blended with 5CB contained less distinct features in the phase separation morphology and exhibited a lower value of Sq compared to L,D-PLA, L,D-PLA:SWCN, and L,D-PLA:5CB:SWCN. In the case of Ecoflex^®^, the following trend was found: 35.59 nm (Ecoflex^®^:5CB) > 22.09 nm (Ecoflex^®^:SWCN) > 17.28 nm (Ecoflex^®^) > 14.91 nm (Ecoflex^®^:5CB:SWCN). On the other hand, the mixture of PCL with additives showed a slightly different behavior (see Table 2), which can probably be explained by the different miscibilities of all polymers with one or two additives. 

In summary, the Sq parameter expresses the root mean square of deviations from the ideal plane, so it can be assumed that for the created layers, the addition of 5CB does not change the surface roughness (the order of magnitude Sq does not change, except PCL). In contrast, the addition of SWCN caused an increase of Sq by one order of magnitude relative to the layer of pure polymer. Therefore, nanotubes increased the surface roughness of composites, and their effect was similar in the samples with and without the 5CB dopant.

### 3.2. UV–Vis Studies

The optical properties of polymers and hybrid compositions were investigated in chloroform solution and in thin film by UV–Vis absorption spectroscopy. First, we investigated three biodegradable polymers in chloroform by UV–Vis spectroscopy at various concentrations, monitoring the changes in the π–π transitions. For L,D-PLA and PCL with concentrations of 500 mg/mL, the absorption curves were investigated for solutions diluted about 25-times, while for Ecoflex^®^, it was necessary to dilute the solution 25,000 times. The UV–Vis absorption spectra of the polymers together with the calibration curves are presented in Figure S5. Our study showed that with an increase in the concentration of all investigated polymers, we did not observe changes in the maximum of the absorption band. All investigated polymers exhibited one main absorption band at about 230–240 nm. Moreover, for all investigated polymers, the hyperchromic effect along with increasing concentration was observed. The selected UV–Vis properties of the investigated polymers are presented in Table S1.

**Figure 4:**
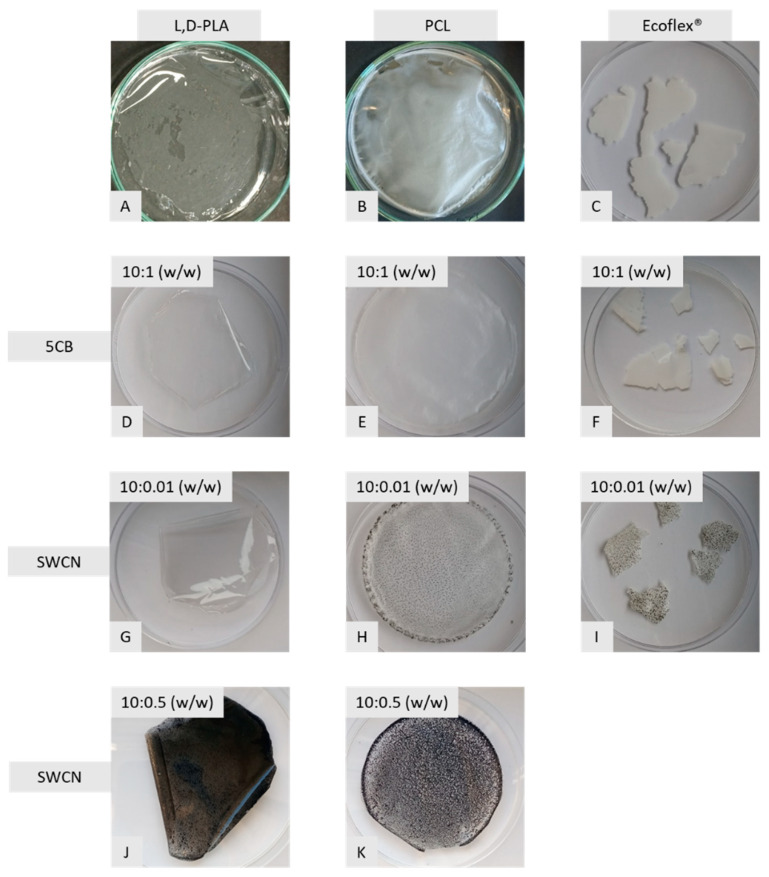
Photos of the created polymer layers by the drop-casting method: polymers L,D-PLA (**A**), PCL (**B**), Ecoflex^®^ (**A**–**C**), polymers with 5CB in a ratio of 10:1 (*w*/*w*) (**D**–**F**), polymers with SWCN in a ratio of 10:0.01 (*w*/*w*) (**G**–**I**), and polymers with SWCN in a ratio of 10:0.5 (*w*/*w*) (**J**,**K**).

In the next step of our work, we investigated the UV–Vis properties of three biodegradable polymers in thin film created by spin-coating and drop-casting techniques. In the spin-coating technique, we used 0.7 mL of polymer solution and created layers with 650 rpm during 60 s. In the drop-cast technique, we used 1 mL of polymer solution. Photos of the created polymer layers are presented in Figure 4 and Figure S6. 

The absorbance and transmittance of the thin created polymer layers are presented in Figure S7. We did not observe big changes in the absorption band of the investigated polymers in the chloroform solution and in thin films. As can be seen from the absorbance spectra for polymers, the absorption bands lay below 300 nm, with the maxima for L,D-PLA at 229 nm, PCL at 239 nm, and Ecoflex^®^ at 266 nm, 284 nm, and 296 nm. Ecoflex^®^ showed the highest absorbance out of all of the investigated polymers. In the range from 350 nm to 1100 nm, L,D-PLA showed the highest transmittance equal to 90%, while the transmittance for PCL oscillated between 20–30% and for Ecoflex^®^ between 20–60%, increasing its value to the highest wavelengths (see Figure S7).

**Figure 5:**
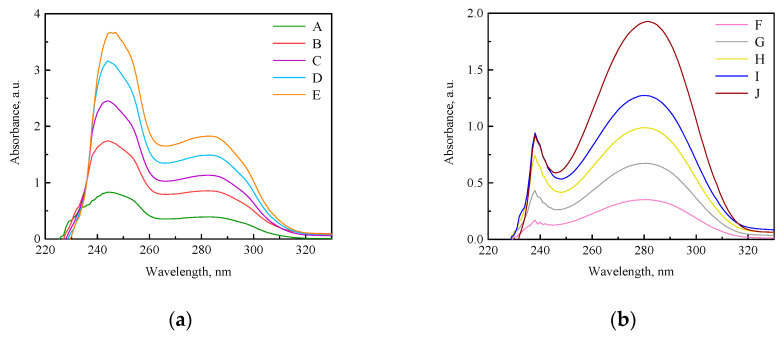
UV–Vis absorption of ternary solutions based on Ecoflex^®^ (**a**) and L,D-PLA (**b**) where A–E is Ecoflex:5CB:SWCN: (A) c (concentration) = 15.2 mg/dm^3^:c = 2.3 mg/dm^3^:c = 8.7 mg/dm^3^; (B) c = 30.3 mg/dm^3^:c = 4.5 mg/dm^3^:c = 17.3 mg/dm^3^; (C) c = 45.5 mg/dm^3^:c = 6.8 mg/dm^3^:c = 26.0 mg/dm^3^; (D) c = 60.6 mg/dm^3^:c = 9.1mg/dm^3^:c = 34.7 mg/dm^3^; (E) c = 75.8 mg/dm^3^:c = 11.3 mg/dm^3^:c = 43.3 mg/dm^3^. (F–J) is L,D-PLA:5CB:SWCN: (F) c = 2106.6 mg/dm^3^:c = 2.3 mg/dm^3^:c = 8.7 mg/dm^3^; (G) c = 4213.2 mg/dm^3^:c = 4.5 mg/dm^3^:c = 17.3 mg/dm^3^; (H) c = 6319.8 mg/dm^3^:c = 6.8 mg/dm^3^:c = 26.0 mg/dm^3^; (I) c = 8426.4 mg/dm^3^:c = 9.1 mg/dm^3^:c = 34.7 mg/dm^3^; (J) c = 10533 mg/dm^3^:c = 11.3mg/dm^3^:c = 43.3 mg/dm^3^.

To check the possibility of using biodegradable polymers with SWCN and 5CB as transparent and conductive anodes in organic devices such as solar cells or light emitting diodes, in the next step, we investigated the optical properties of the binary and ternary mixtures in chloroform solution and as a thin film. Photos of the prepared binary and ternary solutions are presented in Figure S8. UV–Vis spectra of the binary and ternary solutions are presented in Figure 5 and Figure S9 together with the calibration curves. As the weight ratio of 5CB and SWCN increased, no changes in the maximum of the absorption bands of the investigated compositions were observed, only a hyperchromic effect existed. 

**Figure 6:**
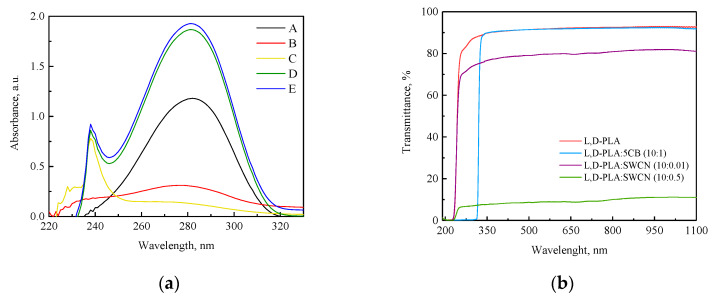
Absorption UV–Vis spectra of L,D-PLA and its compositions, 5CB and SWCN in chloroform with isosbestic points, where A—5CB; B—SWCN; C—L,D-PLA; D—L,D-PLA:5CB (930:1); E—L,D-PLA:5CB (930:1:3.8) (**a**) and the transmittance of L,D-PLA and its compositions (**b**).

For the ternary mixture (polymers and 5CB and SWCN), one isosbestic point was found at about 250 nm (Figure 6a and Figure S9), appearing as an effect of the interactions between two or three compounds.

In the next step, the influence of 5CB and SWCN on the absorption properties of the polymer layers was also determined by UV–Vis spectroscopy (Figure 6b). A general tendency was observed regardless of the polymer used for layer composition. The addition of 5CB in an amount of 1:10 (*w*/*w*) caused a bathochromic shift of both the absorbance and transmittance spectra by 70 nm, reaching a wavelength value of 325 nm, which corresponded to the spectra of 5CB. In the case of L,D-PLA, the presence of 5CB did not cause any additional changes in the spectral properties in the UV–Vis range (Figure 6b). On the other hand, the addition of SWCN in the formed layer increased the baseline in the absorbance spectra and lowered the transmittance by 15%. Moreover, it corresponds well with the amount of used SWCN, the higher the concentration, the greater the influence on the spectral properties of the layer. The addition of SWCN to PCL slightly reduced the transmittance value by about 5% at higher wavelength values. On the other hand, the absorption spectra only demonstrated an increment of the baseline with an increase in the portion of SWCN in the composition. In the case of Ecoflex^®^, the absorption spectra gave an absorbance above 1 given the higher thickness, which made it difficult to analyze. Additionally, the transmittance was found below 5% for all compositions prepared by the drop-casting method. 

Taking into consideration the transmittance of the created layers of the biodegradable polymers with 5CB and SWCN, we can conclude that the 5CB admixture did not change the transmittance of the investigated polymers, while the addition of SWCN reduced the transmittance by approximately 15% for L,D-PLA (see Figure 6b).

### 3.3. Statistic Angle Contact Measurement

Measurements of the static contact angle of the polymer layer through a drop of water have shown that all investigated polymers are hydrophobic, with angle values ranging from 108° to 115° (Table 3). Thus, the application of any aqueous solutions (e.g., PEDOT:PSS) will be problematic in terms of poor interactions and layer formation, as will be shown below. As can be seen from Table 3, the presence of 5CB and SWCN in the film composition caused slight changes in the contact angle value, however, maintained the hydrophobic nature of the films (all the values are above 100°). 

**Figure 7:**
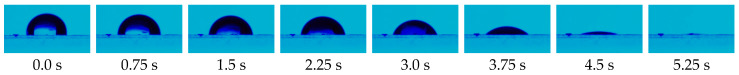
Selected images for the absorption process of water drop by the Ecoflex^®^ layer.

In addition, for the Ecoflex^®^ layers, with and without additives (5CB or SWCN), a rapid sorption of deposited water drop was observed, as it is presented in Figure 7. A visible drop in the volume of droplet was observed for different time periods, starting from the moment the water droplet came into contact with the surface. The droplet spread completely over the film surface after five seconds from deposition. 

### 3.4. Infrared (IR) Thermography Study

**Figure 8:**
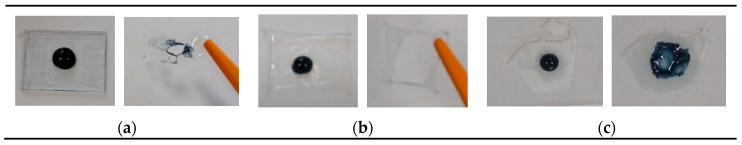
Photo of L,D-PLA (**a**), PCL (**b**) and Ecoflex^®^ (**c**) with poly(3,4-ethylenedioxythiophene):poly(styrenesulfonate) from water (left side) and toluene (right side).

The thermal behavior was studied for films composed of L,D-PLA, PCL and their mixtures with 5CB in ratio 10:1 *w*/*w* (polymer:5CB), and SWCN in ratios of 10:0.01 *w*/*w* or 10:0.5 *w*/*w*. The idea of this study was to assess and select the film with the best electrical properties, allowing its use as a flexible electrode. It was not possible to deposit the poly(3,4-ethylenedioxythiophene):poly(styrenesulfonate) (PEDOT:PSS) solution over our hybrid layers: in the case of a water-based solution, the hydrophobic nature of the surface reduced the possible interaction causing pure or no surface coverage, whereas the use of the toluene-based solution caused the degradation of the composite layers structure (Figure 8).

Therefore, the general architecture of tested devices was a PET/ITO/PEDOT:PSS/PTB7:PC_70_BM(1:1.5)/PFN/Ag/biodegradable polymer film, where L,D-PLA or PCL as the cathode and encapsulated conductive materials were tested. Ecoflex^®^ was not used in this study given the unfavorable mechanical properties enabling handling while constructing the device. In order to ensure the flexibility for all devices, commercial ITO-coated PET (poly(ethylene terephthalate)) was used with a resistance 10^−14^ Ω/square and transparency 70–72%. Resistivity of the PEDOT:PSS was 500–5000 Ω.cm. 

**Figure 9:**
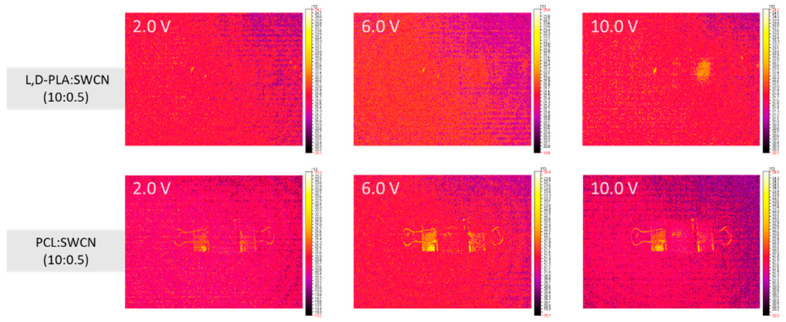
Thermal images for films composed of L,D-PLA, and PCL with SWCN at the ratio 10:0.5 *w*/*w* at 2.0 V, 6.0 V, and 10.0 V.

The tests performed for layers based on L,D-PLA, PCL, L,D-PLA:5CB (10:1), and PCL:5CB (10:1) behaved as electric insulators, as expected. The conductivity appeared when SWCN were present in the polymer film structure. The lowest tested concentration of carbon nanotubes, 10:0.01 *w*/*w* for polymer:SWCN, demonstrated a marginal current passage due to equipment limitations that were unable to correctly assess its accuracy. The resistance for these films was above 100 MΩ, hence no change in the thermal images was noticed (see Figure 9 and Figure S10).

**Figure 10:**
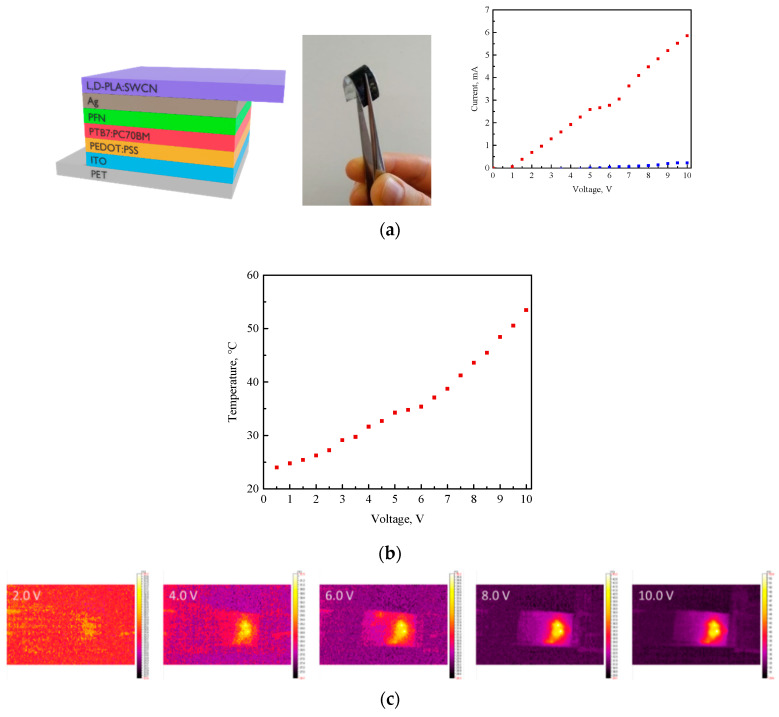
The architecture and photo of the created device and the summary of the thermal experiments for a device composed of PET/ITO/PTB7:PC_70_BM/PFN/Ag/L,D-PLA:SWCN (10:0.5) (red symbols): (**a**) correlation of the current versus applied potential (blue symbols for film LD-PLA:SWCN (10:0.5)); (**b**) correlation of temperature versus applied potential; (**c**) thermal images for devices at 2, 4, 6, 8, and 10 V.

The increase in the SWCN amount from 0.01 to 0.5 significantly improved the conductivity only in the case of L,D-PLA:SWCN (10:0.5), for which above 5 V, a current was measurable. Therefore, only this composition was selected for thermal studies on a device with the architecture of a simple solar cell presented in Figure 10, along with the results obtained for such a device. From the curve current vs. potential, a significant difference in the observed current was noticed when compared to the performance of L,D-PLA:SWCN (10:0.5) alone (see Figure 10a). The constant linear increase of current upon increasing potential confirmed the conductive nature of the whole device, giving the average resistance of 3030.7 Ω. During whole experiment, the highest temperature reached was 53 °C (Figure 10b). 

It should be stressed that in the IR thermography study, we did not use an external source of light. We used an external source of the potential in order to induce thermal response due to current passage to unveil the structural defects. The usage of an external light source could reduce the correct measurement.

## 4. Summary and Conclusions

In this paper, we showed for the first time a comparative study of three different biodegradable polymers (L,D-PLA, PCL, and Ecoflex^®^) in binary and ternary compositions with 5CB or/and SWCN. We investigated the possibility of biodegradable polymer layers as electrodes (anodes and cathodes) in polymer solar cells. For this purpose, we evaluated the conductivity of the hybrid layers consisting of a biodegradable polymer with 5CB in a ratio of 10:1 and polymer:5CB:SWCN in a ratio of 10:1:0.01, and found that both types of layers were transparent but not conductive. With an increase in the amount of SWCN to 0.5 in the composition, the conductivity of the layer only increased for L,D-PLA, losing transparency. 

We also checked the usage of the investigated polymers as a biodegradable substrate instead of PET with the conductive polymer PEDOT:PSS as the anode. Our study showed that all investigated biodegradable polymers were hydrophobic, therefore it was not possible to form PEDOT:PSS layers from toluene or the aqueous solution. Although Ecoflex^®^ exhibited the best hydrophilic properties, it showed the worst film-forming abilities among all of the investigated biodegradable polymers. 

In summary, our study showed that of the three investigated biodegradable polymers, L,D-PLA with the SWCN admixture exhibited the best electrical, optical, and thermal properties for use as part of organic devices (e.g., solar cells, light emitting devices). However, technical modifications and optimization of the layer preparation process are still required to obtain layers of L,D-PLA:SWCN that are more homogeneous and without structural defects. New ideas for SWCN dispersion in a polymer matrix are still needed, because the conductivity and mechanical properties of the hybrid layer depend on it, and hence obtaining a biodegradable, conductive, and flexible polymer layer with a low percolation threshold [16]. 

Finally, the following main conclusions can be formulated:

1. All investigated polymers and hybrid compositions exhibited a semi-crystalline nature with melting/crystallization and glass transition temperatures more or less visible. Addition of SWCN increased the T_g_ for L,D-PLA and Ecoflex^®^. In the case of L,D-PLA, the addition of both 5CB and SWCN decreased the T_g_ compared to the pure polymer and binary compositions. For the Ecoflex^®^:5CB:SWCN compositions, the T_g_ was almost identical as that for the pure polymer. The addition of 5CB to the polymer layer made it more homogeneous with a special track for SWCN and probably simplified the electron transfer in Ecoflex^®^ due to the presence of SWCN.
2. Texture observations by POM showed that the admixture of SWCN had a dominant impact for morphology of the created hybrid layers of all studied polymers. On the other hand, no significant changes in the topography of hybrid layers with or without carbon nanotubes and 5CB were observed for all three polymers studied by the AFM method.
3. No changes in the maximum of absorption bands of the investigated compositions were observed, only the hyperchromic effect with the increase in 5CB and SWCN content.
4. The 5CB admixture did not change the transmittance of the investigated polymers while the addition of SWCN reduced it by approximately 15% in the case of L,D-PLA.
5. It turns out that all investigated polymers were hydrophobic and it was not possible to form PEDOT:PSS layers from toluene or an aqueous solution. Moreover, for the Ecoflex^®^ layers, with and without additives, a rapid sorption of the deposited water drop was observed.
6. The resistance of the polymer:SWCN composition in a ratio of 10:0.01 *w*/*w* was above 100 MΩ, hence no change in the thermal images was noticed. The increase in SWCN content from 0.01 to 0.5 significantly improved the conductivity only in the case of the L,D-PLA:SWCN (10:0.5) composition, for which a current above 5 V was registered with a resistance of 3030.7 Ω.

## Figures and Tables

**Table 1:** Transition temperatures and enthalpy changes of the investigated compositions detected by differential scanning calorimetry (DSC).

Code	Phase Transitions (°C)			
	Heating		Cooling	
	$T_{g}\;(\Delta C_{p}\;\left(\frac{J}{g\cdot {}^{\circ}C}\right))$	$T_{\mathrm{onset}}\;(\Delta H\;\left(\frac{J}{g}\right))$	$T_{g}\;(\Delta C_{p}\;\left(\frac{J}{g\cdot {}^{\circ}C}\right))$	$T_{\mathrm{onset}}\;(\Delta H\;\left(\frac{J}{g}\right))$
L,D-PLA	41.16 (0.35)	137.04 (8.85)	42.45 (0.26)	-
PCL	-	52.69 (61.50)	-	35.80 (−68.104)
Ecoflex^®^	-	-	119.82 (0.54)	-
L,D-PLA:5CB	36.95 (0.29)	135.23 (5.15)	36.88 (0.25)	-
PCL:5CB	-	44.98 (63.21)	-	31.42 (−64.490)
Ecoflex^®^:5CB	-	-	105.38 (0.76)	-
L,D-PLA:SWCN	52.41 (0.38)	140.96 (12.12)	48.42 (0.34)	-
PCL:SWCN	-	53.51 (64.06)	-	45.47 (−63.175)
Ecoflex^®^:SWCN	-	-	126.14 (0.30)	-
L,D-PLA:5CB:SWCN	34.84 (0.28)	132.48 (10.95)	31.45(0.31)	-
PCL:5CB:SWCN	-	46.25 (62.06)	-	40.24 (−61.151)
Ecoflex^®^:5CB:SWCN	-	-	119.72 (0.24)	-

Temperatures in the DSC thermograms obtained during the second heating and cooling cycles at a heating/cooling rate of 10 °C/min.

**Table 2:** The surface parameters of the polymers and hybrid compositions.

Code	Surface Statistics *	
	Sa (nm)	Sq (nm)
L,D-PLA	1.27	2.12
PCL	31.26	43.78
Ecoflex^®^	12.74	17.28
L,D-PLA:5CB	0.80	1.44
PCL:5CB	84.20	109.40
Ecoflex^®^:5CB	25.13	35.59
L,D-PLA:SWCN	13.79	18.63
PCL:SWCN	56.38	75.13
Ecoflex^®^:SWCN	16.97	22.09
L,D-PLA:5CB:SWCN	21.56	28.18
PCL:5CB:SWCN	42.70	55.39
Ecoflex^®^:5CB:SWCN	11.29	14.91

* Values calculated for scanning field 20 µm × 20 µm (400 µm^2^ scan area).

**Table 3:** Results of the contact angle measurements for the polymer layers without and with additives.

Sample	Average Contact Angle, (°)	Image
L,D-PLA	112.9 ± 1.5	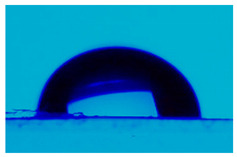
PCL	108.1 ± 0.4	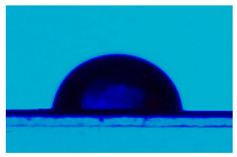
Ecoflex^®^	115.3 ± 2.5	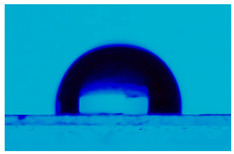
L,D-PLA:5CB (10:1)	111.9 ± 1.6	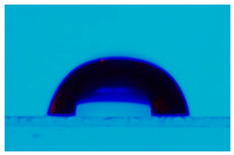
PCL:5CB (10:1)	103.7 ± 0.8	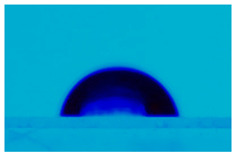
Ecoflex^®^:5CB (10:1)	116.0 ± 0.6	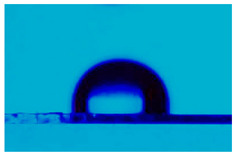
L,D-PLA:SWCN (10:0.01)	105.9 ± 0.2	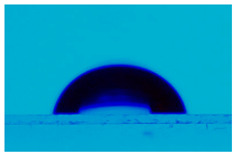
PCL:SWCN (10:0.01)	110.4 ± 0.9	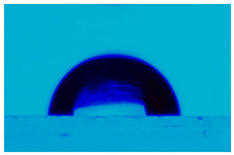
Ecoflex^®^:SWCN (10:0.01)	111.9 ± 0.9	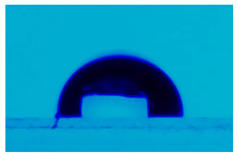

## Supplementary Materials

The following are available online at https://www.mdpi.com/1996-1944/13/11/2480/s1, Figure S1: DSC curves registered during heating and cooling rate of 10 °C/min for created hybrid layers and pure polymers, Figure S2: Optical textures of pure PCL and its hybrid layers registered with crossed polarizes and without analyser, Figure S3: Optical textures of pure Ecoflex^®^ and its hybrid layers registered with crossed polarizes and without analyser, Figure S4: Microscope images of layers with optical magnification of 18×: polymers L,D-PLA, PCL, Ecoflex^®^ (A, B, C), polymers with 5CB in a ratio of 10:1 (*w*/*w*) (D, E, F), polymers with SWCN in a ratio of 10:0.01 (*w*/*w*) (G, H, I) and polymers with SWCN in a ratio of 10:0.5 (*w*/*w*) (J, K), Figure S5: Concentration-dependent solution UV–Vis spectra of the polymers (a, c, e) together with calibration curves (b, d, f)., Figure S6: Photos of polymer layers prepared by the spin-coating method, Figure S7: Absorbance and transmittance of polymer layers of L,D-PLA (a), PCL (b), Ecoflex^®^ (c) created by spin-coating, Figure S8: Photos of polymers (A), polymer:5CB (B), polymer:SWCN (C, D) in chloroform solution, Figure S9: Binary and ternary UV-Vis absorption solutions of polymer:5CB (a, b, c) and polymer:5CB:SWCN (d) compositions in chloroform solution together with calibration curves (e–j). UV-Vis spectra of polymers, 5CB and SWCN in chloroform with isosbestic points (k, l), Figure S10: Thermal images for films composed of on L,D-PLA, PCL, Ecoflex^®^ (a) and their selected mixtures with 5CB or SWCN (b), at 2.0 V, 6.0 V and 10.0V, Table S1: Selected UV-Vis properties of polymers and binary and ternary compositions.
